# BPPO-Based Anion Exchange Membranes for Acid Recovery via Diffusion Dialysis

**DOI:** 10.3390/ma10030266

**Published:** 2017-03-07

**Authors:** Muhammad Imran Khan, Rafael Luque, Pepijn Prinsen, Aziz Ur Rehman, Saima Anjum, Muhammad Nawaz, Aqeela Shaheen, Shagufta Zafar, Mujahid Mustaqeem

**Affiliations:** 1 Department of Chemistry, The Islamia University of Bahawalpur, Bahawalpur 63000, Pakistan; raoimranishaq@gmail.com (M.I.K.); aqeelashaheen1@gmail.com (A.S.), mustaqeem.mujahid@gmail.com (M.M.); 2 School of Chemistry and Material Science, University of Science and Technology China, Hefei 230026, China; 3 Department of Organic Chemistry University of Cordoba, Edificio Marie Curie, Ctra Nnal IV-A, Km396, Córdoba E14014, Spain; q62alsor@uco.es (R.L.); pepijnprinsen33@hotmail.com (P.P.); 4 Department of Chemistry, G.S.C University of Bahawalpur, Bahawalpur 63100, Pakistan; saimabaqer@gmail.com (S.A.); shagutazafar25.sz@gmail.com (S.Z.); 5 Department of Environmental Sciences, Bahauddin Zakariya University, Multan 60000, Pakistan; nawazbzu1@gmail.com

**Keywords:** BPPO, 2-phenylimidazole, anion exchange membrane, diffusion dialysis, acid recovery

## Abstract

To reduce the environmental impact of acids present in various industrial wastes, improved and robust anion exchange membranes (AEMs) are highly desired. Moreover, they should exhibit high retention of salts, fast acid permeation and they should be able to operate with low energy input. In this work, AEMs are prepared using a facile solution-casting from brominated poly-(2,6-dimethyl-1,4-phenylene oxide) (BPPO) and increasing amounts of 2-phenylimidazole (PI). Neither quaternary ammonium salts, nor ionic liquids and silica-containing compounds are involved in the synthesis. The prepared membranes showed an ion exchange capacity of 1.1–1.8 mmol/g, a water uptake of 22%–47%, a linear expansion ratio of 1%–6% and a tensile strength of 0.83–10.20 MPa. These membranes have potential for recovering waste acid via diffusion dialysis, as the acid dialysis coefficient (U_H_) at room temperature for HCl is in the range of 0.006–0.018 m/h while the separation factor (S) is in the range of 16–28, which are higher than commercial DF-120B membranes (U_H_ = 0.004 m/h, S = 24).

## 1. Introduction

Ion exchange membranes can be suitable for diffusion dialysis (DD) processes [1,2,3]. The membranes should have a sufficient water uptake and ion exchange capacity [4,5]. DD is driven by a concentration gradient and is an environmentally friendly downstream technology to retrieve acids and alkali from waste solutions, which often contain metal ions from steel production, metal refining, electroplating, resin regeneration and aluminum etching [6,7,8]. For instance, in the pickling process for metal surface treatment, sulfuric–, hydrochloric– and nitric–hydrofluoric acid or mixtures of them are used [9]. Advantages of the DD process are low energy consumption, relatively simple operation and higher separation efficiency compared to other treatment methods such as solvent extraction, crystallization, neutralization and thermal decomposition. The acid recovery depends on the equilibrium concentration, hydrodynamic conditions and mode of operation. Furthermore, the efficiency and long-term stability depend largely on the ion exchange membrane properties.

Anion exchange membranes can be used for a more cost-effective recovery of acids [10,11], among other membrane-based applications. In the acid recovery process, the membrane should possess high acidic resistance and good anion and proton permeability while, at the same, time retaining the metal cations in the retentate; it should also possess low salt and water permeability [8,9], to recover the acid in a more cost-effective way. Most of the AEMs (anion exchange membranes) used for acid recovery are homogeneous and are fabricated from different polymers. The polymer main chain of AEMs must be hydrophobic and mechanically robust to attain high stability. To obtain AEMs with a high diffusion coefficient, the ion exchange capacity (IEC) must be high enough, and it can be attained by the presence of well-connected ion conducting channels [2]. However, the attachment of functional groups to the polymer backbone may lead to lower selectivity between protons and other cations. Moreover, excessive swelling and poor chemical stability are also drawbacks of AEMs with a high IEC. Among various AEM polymer backbones, poly-(2,6-dimethylene-1,4-phenylene oxide) (PPO) is extensively used for its excellent mechanical properties and facile membrane fabrication after treatment with bromine (BPPO). Alternatively, commercially available BPPO can be directly used as a precursor of a wide variety of AEMs, avoiding the commonly employed chloromethylation method which uses highly toxic chloromethyl ether (CME) [12].

Previously, we reported the fabrication of methylpyrrolidine (MP)-based AEMs for acid recovery by DD [4]. However, MP has a relatively low acidity (pK_a_ = 10.32), resulting in rather low anion permeability. In the current work, a series of AEMs are synthesized from commercial BPPO by increasing the amounts of phenylimidazole (PI, pK_a_ = 6.5). These membranes show higher acid stability due to the non-aromatic character of PI, which may also be beneficial for electrochemical applications. The prepared membranes were evaluated using FTIR (Fourier Transform Infrared) spectroscopy, thermal stability (TGA), ion exchange capacity (IEC), water uptake (WU), the linear expansion ratio (LER), morphology (SEM), and chemical and mechanical stability. Finally, they were tested for acid recovery via DD in a feed mixture containing HCl and FeCl_2_.

## 2. Experimental

### 2.1. Materials

Brominated poly-(2,6-dimethyl-1,4-phenyleneoxide) (BPPO) and commercial DF-120B anion exchange membranes were supplied by Tianwei Membrane Co. Ltd. (Weifang, China). DF-120B are manufactured from quaternary ammonium poly-(2,6-dimethyl-1,4-phenylene oxide) (QPPO) with polyester as a substrate. The 2-phenylimidazole (PI) was obtained from Sinopharm Chemical Reagent Co. Ltd. (Beijing, China). All other reagents used during the experiments were of analytical grade and commercially available from domestic chemical reagents companies and used without further purification. Deionized water was used in all the experiments.

### 2.2. Membrane Preparation

**Figure 1:**
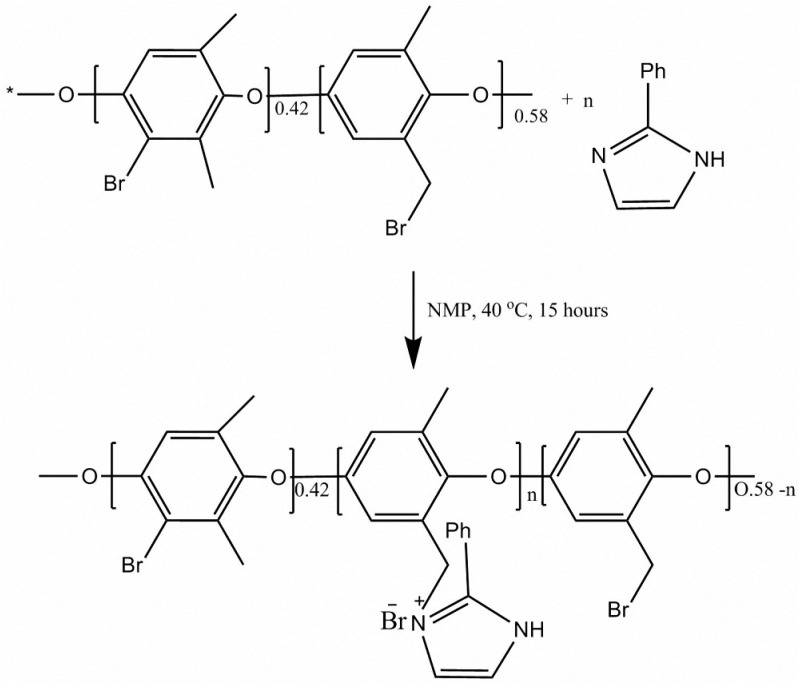
Synthesis of brominated poly-(2,6-dimethyl-1,4-phenyleneoxide) (BPPO)-based anion exchange membranes.

The membranes were prepared using the solution casting method. The casting solution composed of 4% brominated poly-(2,6-dimethyl-1,4-phenylene oxide) (BPPO) in N-methyl-2-pyrrolidone (NMP) solvent. AEMs were prepared by adding 6, 9, 11 and 13 wt % of 2-phenylimidazole (PI) in the casting solution, denoted as PI-6, PI-9, PI-11 and PI-13 membranes. The casting solution was stirred vigorously for 15 h at 40 °C. Finally the solution was casted onto a glass plate and heated for 24 h at 60 °C to remove the solvent. The AEMs were peeled off from the glass plates and washed with deionized water before further use. Figure 1 resumes the chemical synthesis of the prepared AEMs.

### 2.3. Characterization

#### 2.3.1. Functional Groups and Thermal Stability

Fourier Transform Infrared Spectrometry (FTIR) was used to study the presence of functional groups. Spectra of the dried membranes were recorded with the help of an FTIR spectrometer (Vector 22, Bruker, Massachusetts, MA, USA), following the attenuated total reflectance (ATR) with a resolution of 2 cm^−1^ and a total spectral range of 400–4000 cm^−1^. Thermogravimetric analysis (TGA) was used to evaluate the thermal stability using a Shimadzu TGA-50H (Shimadzu Corporation, Kyoto, Japan) within the temperature range 25–700 °C in dynamic nitrogen atmosphere with a heating rate of 10 °C·min^−1^.

#### 2.3.2. Morphology

Morphological characterization was carried out using a field emission scanning electron microscope (FE-SEM, Sirion200, FEI Company, Hillsboro, OR, USA). Surface and cross-sectional views were taken from dry membranes.

#### 2.3.3. Ion Exchange Capacity (IEC)

The IEC represents the number of exchangeable ionic group equivalents present per dry membrane weight. IECs were measured by the classical Mohr method [5,13,14]. First, the membrane samples were equilibrated in 1.0 M NaCl solution for two days such that all charge sites were converted to Cl^−^ salts. Then, the membranes were washed carefully with deionized water to remove excess NaCl. The washed membranes were then equilibrated with 0.5 M Na_2_SO_4_ solutions for two days. The amount of Cl^−^ ions liberated was estimated by titration with 0.05 M AgNO_3_ using K_2_CrO_4_ as an indicator. The IEC (mmol/g) of the membrane was calculated by the equation “IEC = *VC*/*m*” where *m*, *V* and *C* represents the membrane dry weight, the titre value and the AgNO_3_ concentration, respectively.

#### 2.3.4. Water Uptake, Linear Expansion Ratio and Thickness

The hydrophilic nature of the membranes was studied by water uptake (WU) measurements. Membrane samples were oven dried to dry weight. Then, the membranes were immersed in water for 72 h at 25 °C and the wet weight of those membranes were recorded after removal of surface water with tissue paper. WU values were calculated [5,13,14,15] as the weight gain per gram of the dry sample using Equation (1).

(1) $$\mathrm{WU}=\frac{W_{\mathrm{WET}}-W_{\mathrm{DRY}}}{W_{\mathrm{DRY}}}\times 100\%$$

*W*_WET_ and *W*_DRY_ are the weights of wet and dry membrane, respectively. The linear expansion ratio (LER) was measured using 2 × 2 cm^2^ membrane pieces at 25 °C. The LER is calculated with the following equation [5,13,14,15]:

(2) $$\mathrm{LER}=\frac{\left(L_{\mathrm{WET}}-L_{\mathrm{DRY}}\right)}{L_{\mathrm{DRY}}}\times 100\%$$

*L*_WET_ and *L*_DRY_ are the lengths of wet and dry membranes, respectively. The thickness of dry anion exchange membranes was measured carefully using a micrometer screw gauge.

#### 2.3.5. Chemical and Mechanical Stability

The acid stability was assessed by immersing the dry membrane samples in 2 M HCl at ambient temperature for 72 h. The samples were then taken out and washed thoroughly with deionized waters. The percentage of weight loss during this procedure was considered as the acid stability. Tensile strengths (TS) of the membranes were measured at room temperature in wet state using a Q800 dynamic mechanical analyzer (DMA, TA Instruments, Kyoto, Japan) at a stretch rate of 0.5 N/min.

#### 2.3.6. Diffusion Dialysis of HCl/FeCl_2_ Mixtures

Diffusion dialysis (DD) tests were performed in a two-compartment cell of equal volume separated by the membrane with an effective area of 5.7 cm^2^. Prior to testing, all the membranes were conditioned carefully in the feed solution (0.81 M HCl + 0.18 M FeCl_2_) for 2 h, which resembles a waste acid solution produced from metallurgical processes. Then, one compartment of the cell is filled up with 100 mL feed solution while the other side with 100 mL water. In order to minimize the concentration polarization, both sides were stirred vigorously. Diffusion was allowed to progress for 1 h. After removing feed and permeate from the different compartments, HCl concentrations of both sides were determined by titration with aqueous Na_2_CO_3_ solution (0.05 M), while FeCl_2_ concentrations were determined by titration with aqueous KMnO_4_ solution (0.002 M). All the experiments were conducted at room 25 °C. The dialysis coefficients U (m/h) can be calculated by using Equation (3) [13,15]:

(3) $$U=\frac{M}{At\Delta C}$$

*M* is the amount of diffused compound (mol), *A* is the effective membrane area (m^2^), *t* is the time (h), and ∆*C* is the logarithm average concentration difference between the two chambers (mol·m^−3^). ∆*C* is calculated as below [13,15]:

(4) $$\Delta C=\frac{C_{f}^{0}-(C_{f}^{t}-C_{d}^{t})}{\ln[C_{f}^{0}/(C_{f}^{t}-C_{d}^{t})]}$$

$C_{f}^{0}$ and $C_{f}^{t}$ are feed concentrations at time 0 and *t*, respectively, and $C_{d}^{t}$ is the dialysate concentration. U_H_ and *U*_Fe_ are calculated according to Equations (3) and (4). The separation factor (*S*) is defined as the ratio of dialysis coefficients of the two species present in the solution and can be calculated as [13,15]:

(5) $$S=\frac{U_{H}}{U_{\mathrm{Fe}}}$$

## 3. Results

### 3.1. Functional Groups

**Figure 2:**
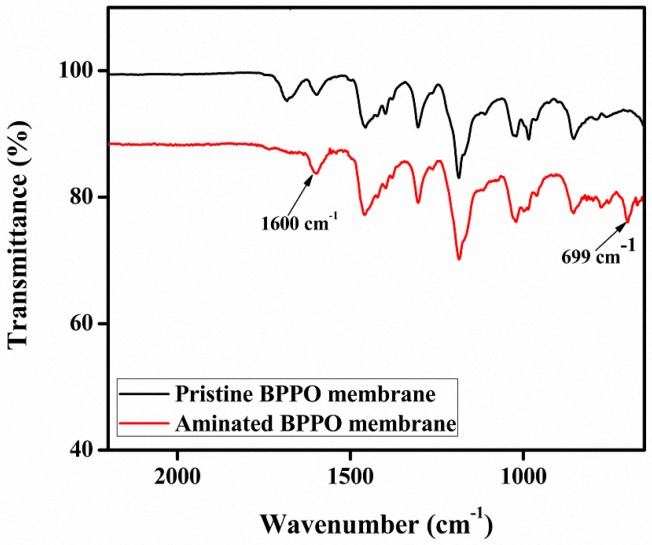
IR spectrum of BPPO-based AEMs.

The successful synthesis of BPPO-based AEMs was confirmed by FTIR (Figure 2). The reference band around 1450 cm^−1^ originated from the CH stretching vibrations (V and δ) present in BPPO as well as the aminated BPPO [16]. The band observed at 1600 cm^−1^ in the aminated BPPO membrane confirmed the stretching vibration of C–N which is absent in pristine BPPO membranes. The adsorption peaks of the symmetrical and asymmetrical stretching vibrations of C–O are at 1200 cm^−1^ and 1310 cm^−1^, and those of phenyl group are at 1470 cm^−1^ and 1600 cm^−1^, respectively. The presence of the two sharp, intense peaks around 760 cm^−1^ and 700 cm^−1^ is due to the pendant benzene rings in aminated BPPO [17], which clearly suggest that the reaction proceeded successfully as they were absent in the spectrum of the pristine BPPO membrane.

### 3.2. Thermal Stability

**Figure 3:**
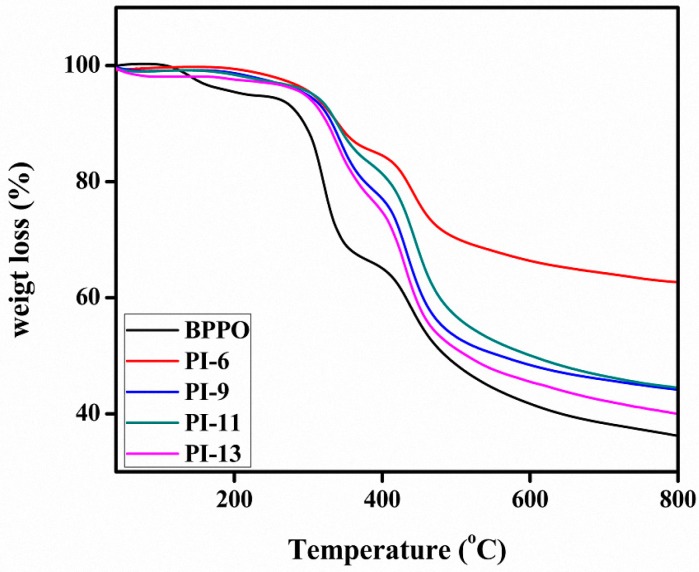
TGA thermograms of BPPO-based AEMs.

The thermal stability of the prepared membranes was evaluated with thermogravimetric analysis (TGA) as shown in Figure 3. The prepared membranes showed good thermal stability in the medium temperature range. The weight loss occurred in three main stages: desorption of water, thermal deamination and thermal oxidation. Water loss due to the evaporation of bound water from the membrane matrix occurred in the 80–150 °C range, followed by significant weight loss starting around 300 °C which is possibly due to the degradation of the quaternary ammonium groups [10]. The final weight loss occurred starting from 420 °C, caused by the degradation of the main polymer backbone.

Table 1 shows the thermal decomposition temperature (T_d_) and the initial decomposition temperature (IDT) of the prepared membranes. PI-6, PI-9, PI-11 and PI-13 have an IDT in the range of 285–294 °C and a Td in the range of 303–311 °C, whereas the values of the pristine BPPO membrane are 185 °C and 225 °C, respectively [13]. The results demonstrate the higher thermal stability of the prepared AEMs compared to the previously prepared ones [13].

### 3.3. Ion Exchange Capacity (IEC), Water Uptake (WU) and Linear Expansion Ratio (LER)

The IEC of AEMs is a measure of the density of the dissociable ionic groups in the membrane matrix, which are responsible for ion transport across the membrane [13]. The IEC values of the prepared AEMs were estimated by a titration method (Table 2). The IEC increased from 1.14 to 1.78 mmol/g when doubling the amount of PI added in the membrane synthesis (PI-6 vs. PI-13), which confirms an increase in the hydrophilicity of the membrane. In any case, the IEC values exceeded the ion exchange capacity of the commercial membrane DF-120B (0.83 mmol/g). The WU is also an important parameter for hydrophilicity, and it influences separation phenomena, dimensional aspects as well as mechanical properties [5,18,19,20,21]. Dissociation of the charged functional groups could be promoted by the presence of water molecules inside the membrane matrix and they are very crucial for the transportation of ions [5,21]. However, higher water uptake generally undermines the selectivity [22]. The WU increased from 22% to 47% with higher amounts of PI (Table 2), but stayed below the value of 74% of the commercial DF-120B membrane and even below the advanced hierarchical porous AEMs prepared with quaternized amino-ethanol–derived PPO (QAPPO) and organosilane ionic liquids (51%–158%) [22]. Thus, the insertion of PI units increased the presence of hydrophilic regions in the membrane matrix in a substantial way and this can be used to control the water uptake. Finally, the linear expansion ratio (LER) was determined (Table 2). The LER values increased from 1% to only a maximum of 6% with increasing amounts of PI in the membrane matrix, which is relatively low, compared to the above-mentioned QAPPO-based AEMs (3%–8%) [22]. It improved the swelling resistance and enabled longer membrane operational times. The thickness of the prepared anion exchange membranes was found to vary randomly between 17–31 μm, which was thinner than QAPPO-based AEMs (43–60 μm) [22], which may increase the tortuosity of ion transfer to improve the selectivity of the membranes and enhance the mass transport as well. The thickness of the prepared membranes was found to be different between them because no specific technique was used to attain a uniform membrane thickness, and this could therefore be an extra variable in the diffusion performance of the different membranes.

### 3.4. Chemical and Mechanical Stability

**Figure 4:**
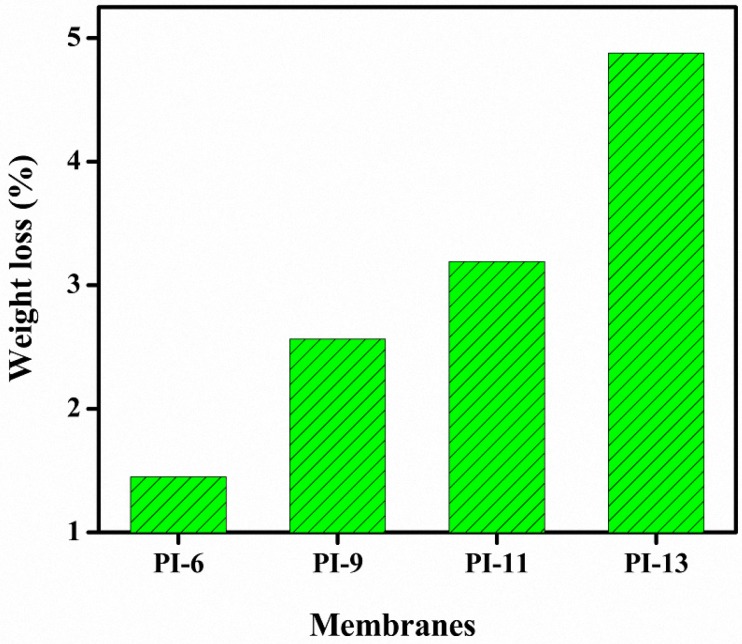
Chemical stability of AEMs with increasing amounts of PI during 72 h in 2 M HCl solution.

The chemical stability of the prepared AEMs is considered as the weight loss after 72 h immersion in 2 M HCl aqueous solution (Figure 4). As the membranes before the chemical stability tests were only washed with deionized water (instead of NaCl buffer), it remained not fully clear if the membrane was completely in its pure cationic form or whether some residual Br anions remained on the imidazole cations in the PI subunits. Therefore, some variation can occur in the chemical stability test due to the anion exchange between Br^−^ and Cl^−^, while the chemical stability test aims to evaluate the loss of functional groups within the polymer material. The possible variations are shown in Table 3 in the function of the PI content and in the function of the residual Br^−^ content after being washed with deionized water. In the case none of the Br ions would have been removed (100 wt % residual Br^−^), the variations are considerable, but for a more realistic content of residual Br^−^ (5 wt %), the effect on the chemical stability test is not significant. The membranes showed acceptable chemical stability and retained their color during the experiment. Moreover, these membranes also seemed to maintain their dimensional integrity, suggesting their suitability in DD processes for acid recovery. However, the chemical instability may become significant with membranes containing amounts higher than 13 wt % PI and with longer periods in the acid. After 144 h weight loss was still observed.

**Figure 5:**
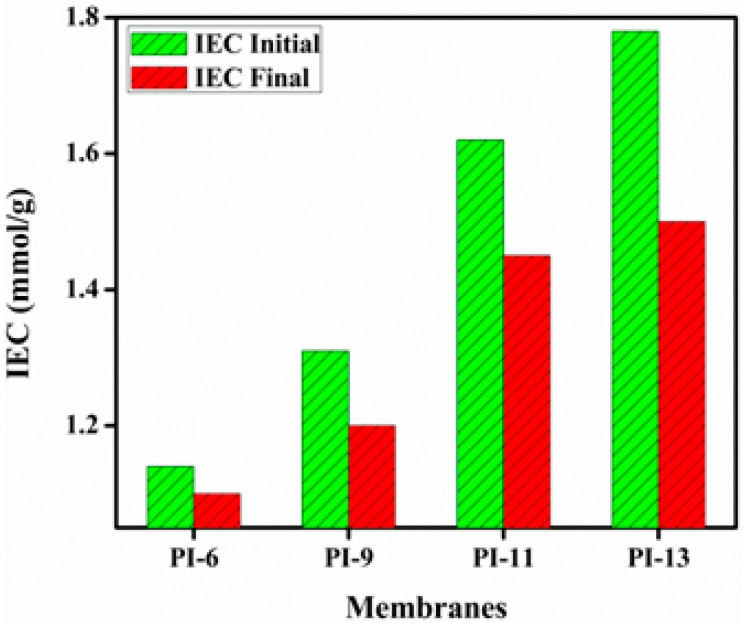
Change in IEC of AEMs after immersion in 2 M HCl for 72 h at room temperature.

The stability of the ion exchange capacity (IEC) of the membranes was also studied in the same experimental conditions as mentioned above to evaluate the chemical stability (Figure 5). The IEC of the membranes was found to be decreased after immersion in 2 M HCl at room temperature. These results are similar to our previous work [5,22]. This is associated with the degradation of the quaternary ammonium group in the membrane matrix [22], and thus in the loss of the retention capacity of ions.

The mechanical stability of the AEMs is also an important operational parameter for their application in acid recovery. It was investigated in wet state at room temperature; the tensile strengths (TS) of the synthesized AEMs are given in Table 2. The TS values of the fabricated AEMs gradually decreased from membrane PI-6 to PI-13 with the increase in the IEC (resulting from the incorporation of ion exchange groups into the initially tight polymer chain network) [22].

### 3.5. Morphology

**Figure 6:**
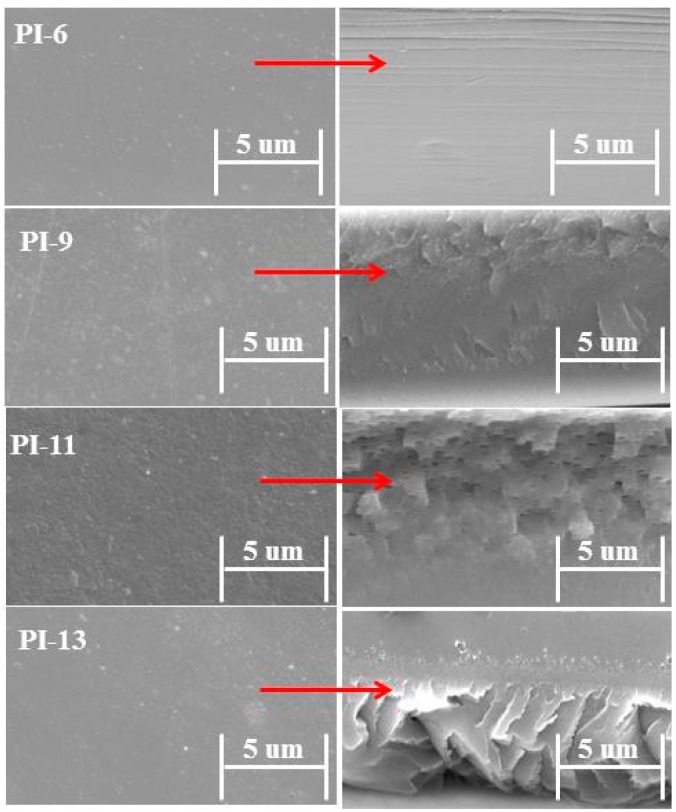
SEM micrographs of surface section (on the left) and cross-section of the AEMs with increasing amounts of PI (on the right).

The morphology of all the prepared membranes is studied by scanning electron microscopy (SEM). The SEM micrographs of the surface and cross-sections are shown in Figure 6. No pores, holes or cracks could be observed in the membrane surface as well as in the cross-section which suggested the homogeneous and dense nature of the prepared membranes. The surface images suggest that better miscibility can be achieved with a higher PI content. No further phase separation was observed when increasing the amount of PI. Therefore, PI can help to control the homogeneity during membrane synthesis.

### 3.6. DD for HCl/FeCl_2_ Solution

**Figure 7:**
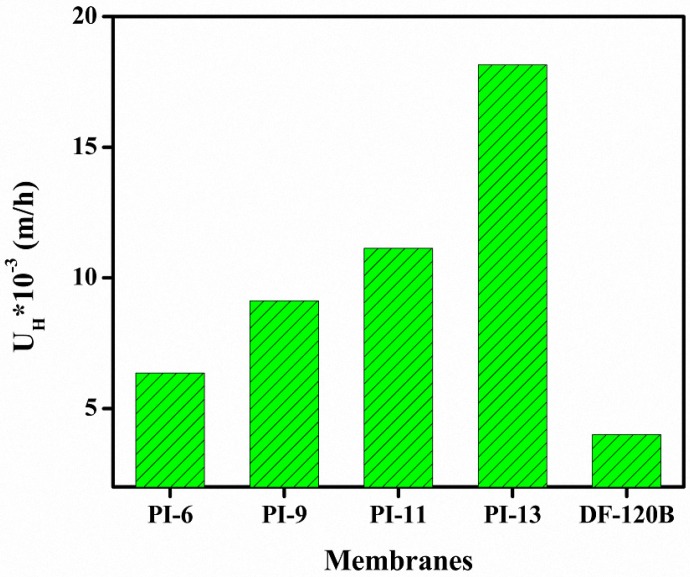
Dialysis coefficient (U_H_) of HCl at 25 °C of BPPO-based AEMs with increasing amounts of PI membranes.

Experiments were performed using mixtures of 0.81 M HCl and 0.18 M FeCl_2_ as a model feed for industrial metal treatment of waste streams to evaluate the potential of the prepared membranes for acid recovery by means of diffusion dialysis (DD). The results are shown in Figure 7. The dialysis coefficient (U_H_) depends on properties such as the ion exchange capacity, water uptake, thickness, binder characteristic, morphology, etc. The U_H_ values of the studied AEMs are in the range of 0.006–0.018 m/h and increase with higher PI content in the membrane matrix. The values are higher compared to the diffusion coefficients of commercial DF-120B membranes (0.004 m/h) and quaternized PPO/SiO_2_ hybrid membranes (0.005–0.011 m/h) [10] and slightly lower than advanced hierarchical porous QAPPO-based AEMs (0.011–0.027 m/h) measured in similar conditions (FeCl_2_:HCl molar ratio 2.2 instead of 2.0) [23]. The prepared AEMs contained imidazole cation groups responsible for the transport of ions across the membranes, which together with other characteristics such as pore size (pore charge density) determined the ion exchange capacity of the membranes. Higher IEC values improved the dialysis efficiency [24,25]. The present ion exchange groups allowed Cl^−^ ions to pass through the membrane concomitantly with H^+^ ions to maintain the electric charge equilibrium. Transport of the Fe-related component (e.g., Fe^2+^ and FeCl^+^) was less likely to occur due to their larger size and lower mobility, which is believed to rely mostly on adsorption phenomena [26]. The results show that the BPPO-based membranes can be potentially employed for the separation of HCl from HCl/FeCl_2_ waste streams.

**Figure 8:**
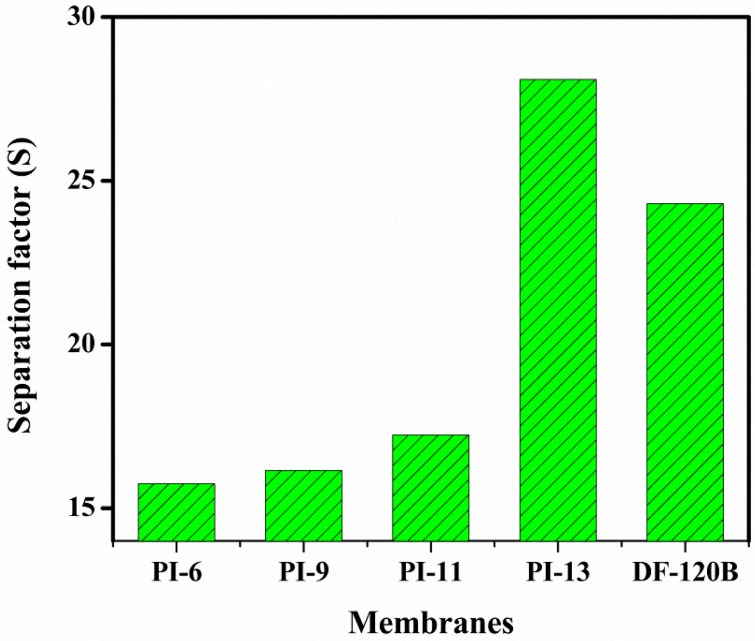
Separation factor (S) for HCl/FeCl_2_ mixtures at 25 °C using BPPO-based AEMs with increasing amounts of PI.

The separation factor S of H relative to Fe, calculated as the ratio of U_H_ to U_Fe_, is represented in Figure 8. The S factor of the prepared AEMs was found to increase from 16 to 28 with increasing amounts of PI in the membrane matrix, in line with the trend for the IEC and water uptake. The PI-13 membrane PI exhibited the highest selectivity (S = 28), which was higher than that of commercial DF-120B membranes (S = 24) [27], but lower than previous results obtained with BPPO-based membranes prepared with multisilicon copolymers (S = 45) [28] and with advanced QABPO-based membranes (S = 39–86) at similar U_H_ values [23]. In many membrane preparation methods, alkyl groups are used in the synthesis to increase the hydrophobicity, which allows the regulation of the selectivity of different anions (compared to Cl^−^ anions in this case) and of different cations (compared to H^+^ in this case) [28]. Hence, the current method regulates the hydrophilicity by incorporation of phenylimidazolium without using quaternary ammonium salts, either expensive ionic liquids or silica-containing compounds.

## 4. Conclusions

In summary, we demonstrate the facile synthesis of anion exchange membranes (AEMs) from commercially available BPPO and 2-phenylimidazole (PI) and its use for acid recovery in waste streams by means of diffusion dialysis (DD). This method permits the fabrication of homogeneous and apparently dense AEMs. With increasing amounts of PI used in AEM synthesis, a higher ion exchange capacity (1.1–1.8 mmol/g) was attained. The water uptake (22%–47%) and linear expansion ratio (1%–6%) increased only substantially when more hydrophilic PI was incorporated in the membrane matrix. The DD results using these membranes showed their potential for HCl separation from Fe-containing waste streams, with dialysis coefficients between 0.006–0.018 m/h and separation factors between 16 and 28. The incorporation of PI segments in the membrane matrix has a positive effect on the separation efficiency during DD.

## Figures and Tables

**Table 1:** Composition, initial decomposition temperature (IDT) and thermal decomposition temperature (T_d_) of brominated poly-(2,6-dimethyl-1,4-phenyleneoxide) (BPPO) anion exchange membranes (AEMs) with increasing amounts of 2-phenylimidazole (PI).

Membranes	PI-6	PI-9	PI-11	PI-13
BPPO (g)	0.4	0.4	0.4	0.4
PI (g)	0.026	0.035	0.043	0.052
IDT (°C)	289	290	294	285
T_d_ (°C)	307	308	311	303

**Table 2:** Ion exchange capacity (IEC), water uptake (WU), linear expansion ratio (LER), thickness and tensile strength of AEMs with increasing amounts of PI.

Membranes	PI-6	PI-9	PI-11	PI-13
IEC (mmol/g)	1.14	1.31	1.62	1.78
WU (%)	22	27	36	47
LER (%)	1.2	2.0	2.3	6.1
Thickness (μm)	17	19	31	20
TS (MPa)	10.2	8.1	2.4	0.8

**Table 3:** Variation (wt %) in the chemical stability tests calculated by function of the residual Br^−^ content after washing with water and by the function of the PI content.

PI Content (wt %)	Residual Br^−^ Content (wt %)			
	100	60	30	5
6	1.3	0.8	0.4	0.1
9	1.9	1.1	0.6	0.1
11	2.3	1.4	0.7	0.1
13	2.6	1.6	0.8	0.1
